# Prevalence of Depression Symptoms in US Adults Before and During the COVID-19 Pandemic

**DOI:** 10.1001/jamanetworkopen.2020.19686

**Published:** 2020-09-02

**Authors:** Catherine K. Ettman, Salma M. Abdalla, Gregory H. Cohen, Laura Sampson, Patrick M. Vivier, Sandro Galea

**Affiliations:** 1 Boston University School of Public Health, Boston, Massachusetts; 2 Brown University School of Public Health, Providence, Rhode Island; 3 Columbia Mailman School of Public Health, New York, New York; 4 Hassenfeld Child Health Innovation Institute, Providence, Rhode Island

## Abstract

**Importance:**

The coronavirus disease 2019 (COVID-19) pandemic and the policies to contain it have been a near ubiquitous exposure in the US with unknown effects on depression symptoms.

**Objective:**

To estimate the prevalence of and risk factors associated with depression symptoms among US adults during vs before the COVID-19 pandemic.

**Design, Setting, and Participants:**

This nationally representative survey study used 2 population-based surveys of US adults aged 18 or older. During COVID-19, estimates were derived from the COVID-19 and Life Stressors Impact on Mental Health and Well-being study, conducted from March 31, 2020, to April 13, 2020. Before COVID-19 estimates were derived from the National Health and Nutrition Examination Survey, conducted from 2017 to 2018. Data were analyzed from April 15 to 20, 2020.

**Exposures:**

The COVID-19 pandemic and outcomes associated with the measures to mitigate it.

**Main Outcomes and Measures:**

Depression symptoms, defined using the Patient Health Questionnaire-9 cutoff of 10 or higher. Categories of depression symptoms were defined as none (score, 0-4), mild (score, 5-9), moderate (score, 10-14), moderately severe (score, 15-19), and severe (score, ≥20).

**Results:**

A total of 1470 participants completed the COVID-19 and Life Stressors Impact on Mental Health and Well-being survey (completion rate, 64.3%), and after removing those with missing data, the final during–COVID-19 sample included 1441 participants (619 participants [43.0%] aged 18-39 years; 723 [50.2%] men; 933 [64.7%] non-Hispanic White). The pre–COVID-19 sample included 5065 participants (1704 participants [37.8%] aged 18-39 years; 2588 [51.4%] women; 1790 [62.9%] non-Hispanic White). Depression symptom prevalence was higher in every category during COVID-19 compared with before (mild: 24.6% [95% CI, 21.8%-27.7%] vs 16.2% [95% CI, 15.1%-17.4%]; moderate: 14.8% [95% CI, 12.6%-17.4%] vs 5.7% [95% CI, 4.8%-6.9%]; moderately severe: 7.9% [95% CI, 6.3%-9.8%] vs 2.1% [95% CI, 1.6%-2.8%]; severe: 5.1% [95% CI, 3.8%-6.9%] vs 0.7% [95% CI, 0.5%-0.9%]). Higher risk of depression symptoms during COVID-19 was associated with having lower income (odds ratio, 2.37 [95% CI, 1.26-4.43]), having less than $5000 in savings (odds ratio, 1.52 [95% CI, 1.02-2.26]), and exposure to more stressors (odds ratio, 3.05 [95% CI, 1.95-4.77]).

**Conclusions and Relevance:**

These findings suggest that prevalence of depression symptoms in the US was more than 3-fold higher during COVID-19 compared with before the COVID-19 pandemic. Individuals with lower social resources, lower economic resources, and greater exposure to stressors (eg, job loss) reported a greater burden of depression symptoms. Post–COVID-19 plans should account for the probable increase in mental illness to come, particularly among at-risk populations.

## Key Points

**Question:**

What is the burden of depression symptoms among US adults during the coronavirus disease 2019 (COVID-19) pandemic compared with before COVID-19, and what are the risk factors associated with depression symptoms?

**Findings:**

In this survey study that included 1441 respondents from during the COVID-19 pandemic and 5065 respondents from before the pandemic, depression symptom prevalence was more than 3-fold higher during the COVID-19 pandemic than before. Lower income, having less than $5000 in savings, and having exposure to more stressors were associated with greater risk of depression symptoms during COVID-19.

**Meaning:**

These findings suggest that there is a high burden of depression symptoms in the US associated with the COVID-19 pandemic and that this burden falls disproportionately on individuals who are already at increased risk.

## Teaser

This survey study examines the prevalence of depression symptoms among US adults during the coronavirus disease 2019 (COVID-19) pandemic compared with before the pandemic.

## Introduction

Coronavirus disease 2019 (COVID-19) and the policies to contain it have been a near ubiquitous exposure for people in the US in 2020. As an event that can cause physical, emotional, and psychological harm, the COVID-19 pandemic can itself be considered a traumatic event.^1^ In addition, the policies created to prevent its spread introduced new life stressors and disrupted daily living for most people in the US. As of April 13, 2020, 42 states were under stay-at-home advisories or shelter-in-place policies, affecting at least 316 million people in the US, or approximately 96% of the population.^2^ The unemployment rate was reaching record highs in the US, with more than 20 million people filing for unemployment between the start of COVID-19 and mid-April 2020.

Mental health is sensitive to traumatic events and their social and economic consequences. Previous studies on disruptions to life owing to disasters, epidemics, or civil unrest suggest that exposure to large-scale traumatic events are associated with increased burden of mental illness in the populations affected.^3^ For example, after September 11, 2001, 9.6% of Manhattan residents reported symptoms consistent with depression and 7.5% reported symptoms consistent with posttraumatic stress disorder.^4^ Residents living closer to the World Trade Centers had higher prevalence of mental illness.^4,5^ Similarly, after natural disasters, populations affected by hurricanes report an increase in symptoms consistent with mental illness.^3^ Increases in mental illness have also been documented after other epidemics, such as the Ebola virus and SARS outbreak.^6,7^ In addition, social disruptions in day-to-day living after civil unrest, for example, have been found to be associated with mental illness. Data from Hong Kong show greater levels of depression, anxiety, and psychological distress during the 2019 Hong Kong civil protests.^8,9^

Importantly, the mental health consequences of mass traumatic events are not evenly distributed across populations. Having lower income and less wealth are associated with greater burden of mental illness.^10^ Mental illness has been well documented in the wake of previous financial recessions, particularly among individuals who are unemployed and are otherwise affected by social and economic adversity.^11,12,13,14^

Early evidence from published studies suggests that COVID-19 is associated with mental illness.^15^ Among health care workers in China who were exposed to patients with COVID-19, 50.4% reported symptoms of depression.^15^ A study of medical students in China identified elevated prevalence of anxiety.^16^ Another study found that reduced sleep was associated with greater levels of anxiety and stress among health care workers in China.^17^ To date, most studies regarding mental health and COVID-19 have been conducted in Asia and have focused on specific subpopulations, such as college students^16^ and medical workers.^15,17^ Published studies from the US on mental health have been in purposive samples. Most relevant to this study, a study by Nelson et al^18^ analyzed concerns about COVID-19, symptoms, and responses to the pandemic across 9009 completed surveys distributed over social media. They found that 67.3% of participants were very or extremely concerned about COVID-19 and that 48.8% of participants were self-isolating most of the time to avoid COVID-19. To our knowledge, the mental health of the broader US population during COVID-19 has not been documented.

Aiming to address this gap in understanding, we assessed the burden of depression symptoms in the US during COVID-19 using the same measures deployed in representative national surveys before COVID-19 began. We also aimed to understand the factors associated with depression symptoms during and before COVID-19.

## Methods

This study was approved by the institutional review boards of NORC at the University of Chicago and Boston University. All AmeriSpeak participants provided written informed consent during the enrollment process to join the AmeriSpeak standing panel. All NHANES participants provided written informed consent first for the household interview and then for the health examination. This study followed the American Association for Public Opinion Research (AAPOR) reporting guideline.

### Population

#### Sample During the COVID-19 Pandemic

The primary sample for this study was a nationally representative group of US adults aged 18 years or older using the AmeriSpeak standing panel. The AmeriSpeak panel is a probability-based panel that is representative of the US population by design. Households are randomly selected with a known, nonzero probability from the NORC National Frame, covering approximately 97% of US households. People excluded from the sampling frame were people with PO box–only addresses, some addresses not listed in the US Postal Service Delivery Sequence File, and some newly constructed homes. Adults aged 18 years or older who could speak English and who had completed an AmeriSpeak survey in the past 6 months were eligible to take the survey. In total, 1470 participants completed the survey, representing a survey completion rate of 64.3% of sampled panelists. The survey was conducted mainly over the internet (1385 participants [94.2%]) with a small number conducted over the telephone (85 participants [5.8%]). Twenty-nine participants missing data for depression were excluded from the sample. The final COVID-19 study sample included 1441 participants.

The survey was distributed by NORC at the University of Chicago from March 31, 2020, through April 13, 2020, assessing COVID-19 exposure, stressors, and mental health using the COVID-19 and Life Stressors Impact on Mental Health and Well-being (CLIMB) study questionnaire. Participants were contacted via web survey and follow-up was conducted via telephone interview. Several demographic questions (eg, sex, age, self-reported race/ethnicity, educational status, and marital status) were previously assessed for all AmeriSpeak panel members. As members of the AmeriSpeak panel, participants are invited to participate in several surveys per month and were paid a cash equivalent of $3 for completing this survey.

#### Comparison Sample Before the COVID-19 Pandemic

The comparison sample for this study, measuring mental health before COVID-19, was the National Health and Nutrition Examination Survey (NHANES), a nationally representative group of noninstitutionalized civilian US adults aged 18 years or older. The NHANES is an annual cross-sectional survey conducted by the US government. Participants are selected through a 4-stage probability sampling design, selecting primary sampling units by the county-level, then by census block–level, and then by households in the 50 states and the District of Columbia. The AmeriSpeak panel’s sampling frame included 97% of US households, in all 50 states and the District of Columbia, and used a 2-stage probability sample design, first at the county-level and then at the census block–level. Thus, in this way, the NHANES sample is a suitable comparison group for the nationally representative AmeriSpeak panel. Collection of NHANES is administered through household interviews and interviews in a mobile examination center, measuring physical and mental health with a Computer Assisted Personal Interview for the depression screener, similar to an online survey questionnaire. There were 9254 participants in the NHANES 2017 to 2018 cycle. The NHANES sample used in this study excluded 3398 individuals (36.7%) younger than 18 years and 791 individuals (8.5%) missing depression data. The final NHANES sample included 5065 participants.

### Key Definitions

#### Depression Symptoms

Depression symptoms in both studies were assessed using the Patient Health Questionnaire–9, a clinically validated survey with a sensitivity of 88% and a specificity of 88% at a cutoff score of 10 or higher.^19^ Depression symptom categories were defined as none (score, 0-4), mild (score, 5-9), moderate (score, 10-14), moderately severe (score, 15-19), and severe (score, ≥20).^19^ Binary classification of depression symptoms was defined by a score of 10 or greater.

#### COVID-19 Stressors Score

We assessed 13 stressors based on prior studies conducted after traumatic events.^20,21^ Examples of COVID-19 stressors included losing a job, death of someone close to you owing to COVID-19, and having financial problems. We excluded stressors that were capturing constructs applicable only to specific groups and created a score ranging from 0 to 13 to measure cumulative exposure to COVID-19 stressors. We divided the scores into thirds to measure low, medium, and high exposure to COVID-19-induced stressors. Cutoffs for stressor score categories were low (score, 0-2), medium (score, 3-4), and high (score, 5-13).

#### Demographic Characteristics

Sex was defined as a binary variable for men or women. Age was defined as a categorical variable with 3 groups: 18 to 39 years, 40 to 59 years, or 60 years or older. Race/ethnicity was defined as a categorical variable across 5 mutually exclusive categories: non-Hispanic White, non-Hispanic Black, Hispanic, non-Hispanic Asian, and other race/ethnicity (including multiple races/ethnicities). Education was defined as a categorical variable with 4 groups: less than high school graduate, high school graduate or general education diploma equivalent, some college, and college graduate or higher. Marital status was defined as a categorical variable with 4 groups: married; widowed, divorced, or separated; never married; and living with partner. Household income was defined as a categorical variable with 4 groups, divided approximately at the interquartile range: $0 to $19 999, $20 000 to $44 999, $45 000 to $74 999, and $75 000 or more. Household savings was defined as a binary variable with savings of at least $5000. Savings was defined as “money in all types of accounts, including cash, savings, or checking accounts, stocks, bonds, mutual funds, retirement funds (such as pensions, IRAs, 401Ks, etc), and certificates of deposit.” Household size was defined as the number of people living in a home with categories from 1 to 7 or more to protect participant identity.

### Statistical Analysis

First, we calculated the demographic characteristics of the NHANES and CLIMB samples. Mobile examination center survey weights were used for NHANES data, and probability survey weights were used for CLIMB data. Second, we conducted bivariable χ^2^ analysis to assess the association between demographic characteristics and depression symptoms in CLIMB and NHANES samples. Third, we estimated the prevalence and 95% CIs of depression symptoms in the US across categories before and during COVID-19 using CLIMB and NHANES samples. Fourth, we calculated the difference and ratio between estimates of depression symptoms during and before COVID-19. Fifth, we estimated the distribution of depression symptom categories before and during COVID-19 in the US using CLIMB and NHANES samples. Sixth, we used multivariable logistic regression to estimate odds ratios (ORs) and 95% CIs for the association between COVID-19–induced life stressors and depression symptoms, controlling for demographic characteristics and resources; the model controlled for sex, age, race/ethnicity, household size, education, marital status, household income, household savings, and COVID-19 stressor score. We used complete case analysis for the multivariable analysis. Stata statistical software version 16.1 (StataCorp) was used for statistical analyses. *P* values were 2-sided, and statistical significance was set at *P* = .05. Data were analyzed from April 15 to 20, 2020.

## Results

A total of 1470 participants completed the CLIMB survey for a completion rate of 64.3%. Of these, 1441 participants were included in the final sample. A total of 619 participants (43.0% unweighted, 38.0% weighted) in the total sample were aged 18 to 39 years, 723 (50.2% unweighted, 48.1% weighted) were men, and 933 (64.7% unweighted, 62.9% weighted) were non-Hispanic White. The pre–COVID-19 NHANES sample included 5065 participants (1704 participants [37.8%] aged 18-39 years; 2588 [51.4%] women; 1790 [62.9%] non-Hispanic White). Table 1 presents demographic characteristics of the NHANES and CLIMB study participants, prevalence of depression symptoms for each sample weighted to the US population, and distribution of depression symptoms by demographic groups. Our sample was representative of the US population and similar in distribution of demographic characteristics to that of the NHANES sample. The CLIMB sample had a lower income than the NHANES sample (Table 1).

**Table 1.** Demographic Characteristics of Nationally Representative Samples of US Adults Before and After the COVID-19 Pandemic and Association With Depression Symptoms

Characteristic	Before COVID-19^a^			During COVID-19^b^		
	No. (%)^c^		*P* value^e^	No. (%)^c^		*P* value^e^
	Total (n = 5065)	Depression symptoms (n = 458)^d^		Total (n = 1441)	Depression symptoms (n = 382)^d^	
Sex						
Men	2477 (48.6)	181 (6.9)	.02	723 (48.1)	149 (21.9)	<.001
Women	2588 (51.4)	277 (10.1)		718 (51.9)	233 (33.3)	
Age, y						
18-39	1704 (37.8)	149 (9.0)	.62	619 (38.0)	219 (38.8)	<.001
40-59	1542 (34.2)	144 (8.5)		462 (32.4)	113 (26.8)	
≥60	1819 (28.0)	165 (7.9)		360 (29.7)	50 (14.9)	
Race/ethnicity						
Non-Hispanic White	1790 (62.9)	186 (8.4)	.01	933 (62.9)	225 (26.5)	.32
Non-Hispanic Black	1176 (11.1)	97 (8.4)		143 (11.9)	36 (24.2)	
Hispanic	1155 (15.8)	109 (8.4)		255 (16.6)	84 (34.0)	
Non-Hispanic Asian	674 (5.3)	26 (4.4)		36 (3.1)	8 (23.1)	
Other or multiple	270 (4.8)	40 (16.0)		74 (5.6)	29 (34.4)	
Education						
<High school	958 (11.0)	117 (13.3)	.004	65 (9.8)	22 (29.2)	<.001
High school or GED	1292 (28.4)	129 (9.3)		274 (27.9)	85 (35.0)	
Some college	1636 (30.5)	158 (9.8)		637 (27.8)	186 (32.0)	
≥College	1173 (30.1)	52 (4.7)		465 (34.5)	89 (18.3)	
Marital status						
Married	2404 (53.4)	146 (5.5)	<.001	712 (47.8)	124 (18.3)	<.001
Widowed, divorced, or separated	1093 (18.5)	156 (13.9)		247 (18.4)	75 (31.5)	
Never married	870 (18.7)	91 (10.5)		344 (24.2)	130 (39.8)	
Living with partner	450 (9.4)	42 (10.7)		138 (9.7)	53 (37.7)	
Household income, $						
≤19 999	868 (12.9)	130 (16.8)	<.001	246 (19.8)	116 (46.9)	<.001
20 000-44 999	1319 (24.0)	133 (10.1)		357 (25.8)	109 (31.1)	
45 000-74 999	887 (19.8)	61 (8.3)		357 (25.1)	83 (23.3)	
≥75 000	1354 (43.4)	68 (4.8)		447 (29.3)	67 (16.9)	
Household savings, $						
≤4999	NA	NA	NA	577 (43.2)	227 (40.4)	<.001
≥5000	NA	NA		819 (56.8)	146 (19.3)	
COVID-19 stressor score^f^						
Low	NA	NA	NA	450 (30.7)	64 (15.5)	<.001
Medium	NA	NA		545 (37.5)	132 (25.1)	
High	NA	NA		443 (31.9)	185 (42.9)	
Household size, mean (SD)	3.1 (0.6)	NA	NA	3.2 (0.6)	NA	NA

Abbreviations: COVID-19, coronavirus disease 2019; GED, general education diploma; NA, not available.

^a^ Before COVID-19 estimates derived from the National Health and Nutrition Examination Survey (NHANES) from 2017 to 2018. Missing data in the NHANES sample included 637 participants with missing household income, 248 participants with missing marital status, and 6 participants with missing education level.

^b^ During COVID-19 estimates derived from the COVID-19 and Life Stressors Impact on Mental Health and Well-being (CLIMB) study collected from March 31 to April 13, 2020. Missing data in the CLIMB study sample included 34 participants with missing household income, 45 participants with missing household savings, and 3 pariticiapnts with missing COVID-19 stressor score.

^c^ Frequencies are unweighted. Percentages are weighted. Categories may not add up to total number owing to missing data. Percentages may not add up to 100 owing to rounding.

^d^ Defined as Patient Health Questionnaire–9 score of 10 or greater.

^e^ Two-tailed χ^2^ analysis conducted for significance testing.

^f^ Calculated from stressor summation (range, 0-13); stratified as low (score, 0-2), medium (score, 3-4), and high (score, 5-13).

A total of 458 participants (8.5%) had depression symptoms before COVID-19, and 382 participants (27.8%) had depression symptoms during COVID-19. Higher levels of depression symptoms were observed in all demographic groups during COVID-19 compared with before, with more than 3-fold higher prevalence of depression symptoms in general. The distribution of depression symptoms within demographic categories was consistent with patterns observed before COVID-19. For example, before and during COVID-19, women were more likely to have depression symptoms than men (before: 277 women [10.1%] vs 181 men [6.9%]; during: 233 women [33.3%] vs 149 men [21.9%]). For race/ethnicity, non-Hispanic Asian individuals saw an 18.7–percentage point higher prevalence of depression symptoms during COVID-19 compared with before COVID-19 (8 participants [23.1%] vs 26 participants [4.4%]).

In general, having more resources was associated with a lower prevalence of depression symptoms, both before and during COVID-19. During COVID-19, married individuals had a lower rate of depression symptoms (124 participants with depression [18.3%]), compared with those who were widowed, divorced, or separated (75 participants with depression [31.5%]), never married (130 participants with depression [39.8%]), or living with a partner (53 participants with depression [37.7%]). Similarly, 116 individuals in the lowest income category (46.9%) had depression symptoms, while 67 individuals in the highest income category (16.9%) had depression symptoms. Individuals with household savings of $5000 or more were less likely to have depression symptoms (146 participants [19.3%]) than individuals with savings less than $5000 (227 participants [40.4%]). Having experienced more COVID-19 stressors was associated with greater burden of depression symptoms (low: 64 participants [15.5%]; medium: 132 participants [25.1%]; high: 185 participants [42.9%]).

Prevalence of depression symptoms in the US was higher in every category during COVID-19 than before COVID-19 (Table 2). During COVID-19, most US residents (52.5% [95% CI, 49.1%-55.8%]) had symptoms of mild depression or greater; before COVID-19, 24.7% (95% CI, 22.9%-26.6%) of US residents had symptoms of mild depression or greater. Mild depression symptoms prevalence was 16.2% (95% CI, 15.1%-17.4%) before COVID-19 compared with 24.6% (95% CI, 21.8%-27.7%) during COVID-19; moderate depression symptoms prevalence was 5.7% (95% CI, 4.8%-6.9%) before COVID-19 compared with 14.8% (95% CI, 12.6%-7.4%) during COVID-19; moderately severe depression symptom prevalence was 2.1% (95% CI, 1.6%-2.8%) before COVID-19 compared with 7.9% (95% CI, 6.3%-9.8%) during COVID-19; and severe depression prevalence was 0.7% (95% CI, 0.5%-0.9%) before COVID-19 compared with 5.1% (95% CI, 3.8%-6.9%) during COVID-19. Overall, prevalence was 1.5-fold higher for mild depression symptoms, 2.6-fold higher for moderate depression symptoms, 3.7-fold higher for moderately severe depression symptoms, and 7.5-fold higher for severe depression symptoms categories during COVID-19 compared with before COVID-19.

**Table 2.** Prevalence of Depression Symptoms in US Adults Before and During the COVID-19 Pandemic

Depression symptoms^a^	% (95% CI)^b^		Difference	
	Before COVID-19^c^	During COVID-19^d^	Absolute, %	Relative
None	75.3 (73.3-77.1)	47.5 (44.2-50.9)	−27.7	0.6
Mild	16.2 (15.1-17.4)	24.6 (21.8-27.7)	8.4	1.5
Moderate	5.7 (4.8-6.9)	14.8 (12.6-17.4)	9.1	2.6
Moderately severe	2.1 (1.6-2.8)	7.9 (6.3-9.8)	5.7	3.7
Severe	0.7 (0.5-0.9)	5.1 (3.8-6.9)	4.4	7.5

Abbrevation: COVID-19, coronavirus disease 2019.

^a^ Depression symptoms assessed using the Patient Health Questionnaire–9 and categorized as none (score, 0-4), mild (score, 5-9), moderate (score, 10-14), moderately severe (score, 15-19), and severe (score, ≥20).

^b^ Percentages weighted to the noninstitutionalized US adult population.

^c^ Before COVID-19 estimates from the National Health and Nutrition Examination Survey from 2017 to 2018.

^d^ During COVID-19 estimates from the COVID-19 and Life Stressors Impact on Mental Health and Well-being study collected from March 31, 2020, to April 13, 2020.

The Figure presents depression symptom scores from the Patient Health Questionnaire–9 grouped by category before and during COVID-19. There was a greater distribution of scores between 0 and 4 before COVID-19; the CLIMB sample had a greater distribution of depression symptoms than the NHANES sample for all scores greater than 5. Thus, we saw a rightward shift in symptom burden in the sample during COVID-19 compared with the sample before COVID-19.

**Figure.**
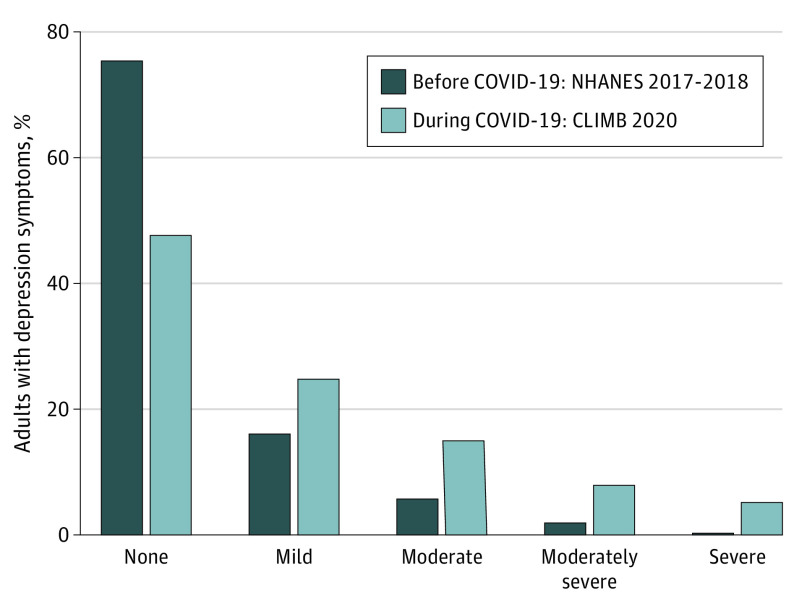
Depression Symptoms in US Adults Before and During the Coronavirus Disease 2019 (COVID-19) Pandemic Before COVID-19 estimates from the National Health and Nutrition Examination Survey (NHANES) from 2017-2018. During COVID-19 estimates from the COVID-19 and Life Stressors Impact on Mental Health and Well-being (CLIMB) study collected from March 31 to April 13, 2020. Depression symptoms categories calculated using the Patient Health Questionnaire–9: none (0-4), mild (5-9), moderate (10-14), moderately severe (15-19), and severe (≥20). Percentages weighted to the population of noninstitutionalized US adults aged 18 years or older.

Among the CLIMB sample, after controlling for sex, age, race/ethnicity, household size, education, marital status, household income, household savings, and COVID-19 stressor score, participants with lower social and economic resources and with higher COVID-19 stressor scores had higher odds of depression symptoms compared with participants with higher social and economic resources or lower COVID-19 stressor scores (Table 3). Compared with married individuals, individuals who were widowed, divorced, or separated had 2.1-fold increased odds of depression symptoms (OR, 2.08 [95% CI, 1.29-3.36]) and individuals who had never married had 1.9-fold increased odds of depression symptoms (OR, 1.85 [95% CI, 1.17-2.94]). Compared with individuals with an annual household income of $75 000 or more, those with a household income of $19 999 or less had 2.4-fold increased odds of depression symptoms (OR, 2.37 [95% CI, 1.26-4.43]). Individuals with household savings less than $5000 had 1.5-fold increased odds of depression symptoms (OR, 1.52 [95% CI, 1.02-2.26]). Experiencing more COVID-19 stressors was also associated with greater odds of depression symptoms compared with people with low stressor exposure (medium: OR, 1.77 [95% CI, 1.16-2.71]); high: OR, 3.05 [95% CI, 1.95-4.77]).

**Table 3.** Odds of Depression Symptoms by Resources and Exposure to COVID-19 Stressors

Resource	OR (95% CI)^a^	*P* value
Education		
<High school	0.86 (0.41-1.77)	.68
High school graduate or GED	1.58 (0.96-2.60)	.08
Some college	1.45 (0.97-2.16)	.07
≥College	1 [Reference]	NA
Marital status		
Married	1 [Reference]	NA
Widowed, divorced, or separated	2.08 (1.29-3.36)	.003
Never married	1.85 (1.17-2.94)	.008
Living with partner	1.27 (0.74-2.18)	.39
Household income, $		
≤19 999	2.37 (1.26-4.43)	.007
20 000-44 999	1.32 (0.78-2.24)	.31
45 000-74 999	1.04 (0.63-1.72)	.88
≥75 000	1 [Reference]	NA
Household savings, $		
≤4999	1.52 (1.02-2.26)	.04
≥5000	1 [Reference]	NA
COVID-19 stressors		
Low	1 [Reference]	NA
Medium	1.77 (1.16-2.71)	.008
High	3.05 (1.95-4.77)	<.001

Abbreviations: COVID-19, coronavirus disease 2019; GED, general education diploma; NA, not applicable; OR, odds ratio.

^a^ Complete case analysis used for multiple logistic regression resulting included 1386 participants for this model. Model controls for demographic characteristics (ie, sex, age, race/ethnicity, and household size). Depression symptoms defined as PHQ-9 score cutoff of 10 or greater. COVID-19 stressor score calculated from stressor summation ranging from 0-13; categories represent low (score, 0-2), medium (score, 3-4), and high (score, 5-13) exposure to stressors due to COVID-19. Data on household income were missing for 34; on household savings, 45 participants; and COVID-19 stressor score, 3 participants.

## Discussion

This survey study found that prevalence of depression symptoms in the US increased more than 3-fold during the COVID-19 pandemic, from 8.5% before COVID-19 to 27.8% during COVID-19. To our knowledge, this is the first nationally representative study that assessed depression symptoms using the Patient Health Questionnaire–9 in US adults before and during the COVID-19-pandemic. We found a shift in depression symptoms, with fewer people with no symptoms and more people with more symptoms during COVID-19 than before COVID-19. We also found that lower income groups were at greater risk of depression symptoms than higher income groups, and that having less than $5000 in household savings was associated with 1.5-fold increased odds of depression symptoms, or 50% greater risk. Additionally, we found that people with exposure to more stressors had greater odds of depression symptoms.

Findings from a 2014 review^3^ on trauma and mental health suggest that depression increases during and after traumatic events; our study adds to this literature. A 2020 study by Ni et al^8^ analyzed depression symptoms before and after political unrest in Hong Kong using the same measure of depression symptoms we deployed in this study. They reported national depression symptoms prevalence before the unrest to be 6.5% (compared with 8.5% in our pre–COVID-19 US sample) and 11.2% in 2019 during unrest (compared with 27.8% in our during–COVID-19 sample). This suggests that the impact of COVID-19 on the US population may be substantially larger than that after other large-scale events. This may reflect the greater ubiquity of COVID-19 and its effects on the US population than prior recorded large-scale traumatic events.

Our findings are consistent with studies in Asia showing a substantial burden of psychological distress following COVID-19.^16,17,22^ In a study of health care workers in Hubei province and surrounding regions, Lai et al^15^ found similar levels of depression: 49.6% of participants had no depression (vs 47.5% of participants in the CLIMB sample), while 35.6% of participants had mild depression (vs 24.6% in the CLIMB sample), 8.6% of participants had moderate depression (vs 14.8% in the CLIMB sample), and 6.2% of participants had moderately severe depression (vs 7.9% in the CLIMB sample). The Lai et al^15^ sample in China included only health care professionals and was concentrated in the Hubei region, while our sample included a representative sample of all US residents and sampled the whole country. Lai et al^15^ sampled earlier in the pandemic, when the global socioeconomic effects of COVID-19 had yet to materialize fully. Our findings are also consistent with a body of literature showing that having fewer assets and more exposure to life stressors are associated with more depression during times of social duress and during low-intensity periods.^10,23,24,25^ Our estimates of pre–COVID-19 depressive symptoms are consistent with other population-level nationally representative studies that captured different constructs of depression; in 2018, 4.7% of adults aged 18 years or older surveyed in the National Survey on Drug Use and Health had major depressive disorder with severe impairment, compared with 8.5% of adults in the NHANES having depression symptoms.^26^ While major depressive disorder is a more severe measure of depression, based on the results of our study, we may expect to see increases in the presence of major depressive disorder in other studies conducted during the COVID-19 pandemic.

### Limitations

This study has some limitations. First, we compare 2 cross-sectional data sources; by comparing NHANES and CLIMB, we are assessing different individuals. We do not survey the same individuals over time, so the NHANES data can provide only a proxy for the true baseline level of depression for CLIMB participants. Differences in prevalence of depression between groups may in part be driven by differences in demographic characteristics, random selection, recruitment, and sampling frame. However, these are both population-representative surveys, drawn randomly with probability-based sampling using address-based sampling of US households, mitigating this concern. We used identical measures of outcome assessment to enable cross-study comparability. Second, some differences in the NHANES and CLIMB depression estimates may be owing to the differences in baseline mental health across participants; for example, estimates of responses to the assessment of previous diagnosis of depression from 2016 suggest that participants in the AmeriSpeak panel had a 2.3–percentage point higher prevalence of previous diagnosis of depression than the national baseline as measured through the Behavioral Risk Factor Surveillance System (18.8% in AmeriSpeak vs 16.5% in the national baseline).^27^ This suggests that the AmeriSpeak population may have had a slightly higher history of previous depression than the national comparison group; however, the difference is small, and our study examined past 2-week depression symptoms. It is impossible to anticipate how the difference in previous depression could, if at all, affect the magnitude of depression found in our study. It is possible that individuals with a known history of depression were more likely to have ongoing depression or have had better access to treatment that would mitigate the current trauma. Third, the COVID-19 pandemic is peaking in different cities at different times; responses to stressors and COVID-19 may differ across regions. This study was not designed to assess regional differences. Fourth, we are using a well-established and validated depression screener. While this provides probably the best possible assessment of population-level burden of depression, a diagnosis of depression is ultimately a diagnosis made by a clinician.

## Conclusions

In this population-representative survey study of US adults, we found that prevalence of depression symptoms was more than 3-fold higher during COVID-19 compared with the most recent population-based estimates of mental health in the US. This increase in depression symptom prevalence is higher than that recorded after previous mass traumatic events, likely reflecting the far more pervasive influence of COVID-19 and its social and economic consequences than other, previously studied mass traumatic events. This study was conducted at the beginning of the COVID-19 pandemic in the US. On the final day of the survey, April 13, 2020, the death toll was more than 23 000 individuals in the US, with more than 600 000 cases confirmed. As of June 8, 2020, the US has experienced more than 113 000 deaths, with more than 2 000 000 cases confirmed. While too few people in the study had been diagnosed with COVID-19 themselves to meaningfully comment on differences in mental health between those who had and had not been diagnosed with COVID-19, we imagine that as the virus spreads and more cases of COVID-19 are confirmed, so too may mental illness increase among those with COVID-19 and those around them. These results suggest that context matters, and that the combined context of the COVID-19 pandemic and its economic consequences have resulted in an increase in mental illness in US adults. Addressing treatment for these individuals will be an interesting and important discussion for health professionals, particularly if a large number of depression cases are due to situational factors. While further data will be needed to assess the trajectory of depression in the US population and potential treatment for affected populations, it seems important to recognize the potential for the mental health consequences of COVID-19 to be large in scale, to recognize that these effects can be long-lasting, and to consider preventative action to help mitigate its effects.^28^ In particular, this burden is being borne by economically and socially marginalized groups, suggesting that individuals with low income and with fewer resources may benefit from particular policy attention in coming months.
